# Further Characterization of an Interleukin-2-1Ike Cytokine Produced by *Xenopus Laevis* T Lymphocytes

**DOI:** 10.1155/1993/68197

**Published:** 1993

**Authors:** Laura Haynes, Nicholas Cohen

**Affiliations:** Department of Microbiology and Immunology University of Rochester Medical Center Rochester New York 14642 USA

**Keywords:** IL-2, *Xenopus*, amphibian, immunity, lymphocyte, cytokine

## Abstract

A T-cell growth factor (TCGF) is produced by antigen- or mitogen-stimulated T lymphocytes from the South African clawed frog *Xenopus laevis*. This study further defines the physical and biological properties of this cytokine and demonstrates that TCGF is biochemically similar to mammalian interleukin-2 (IL-2). Biologically active TCGF eluted from SDS-PAGE displays a M_r_ of 16 kD and lectin-affinity chromatography indicates that the three-dimensionmal configuration of carbohydrates on TCGF and human IL-2 is similar. Secretion of TCGF is detectable 1 day after stimulation of splenocytes with the T-cell mitogen phytohemagglutinin (PHA) and peaks following 2 to 3 days of stimulation. Finally, despite the biological and physical similarities between *Xenopus* TCGF and mammalian IL-2, anti-human IL-2 monoclonal antibodies do not recognize *Xenopus* TCGF.


*Developmental Immunology*, 1993, Vol. 3, pp. 231–238
Reprints available directly from the publisher
Photocopying permitted by license only

© 1993 Harwood Academic Publishers GmbH
Printed in the United States of America

### Further Characterization of an Interleukin-2-like Cytokine Produced by *Xenopus laevis* T Lymphocytes

LAURA HAYNES AND NICHOLAS COHEN*

*Department of Microbiology and Immunology, University of Rochester Medical Center, Rochester, New York 14642*

A T-cell growth factor (TCGF) is produced by antigen- or mitogen-stimulated T lymphocytes from the South African clawed frog *Xenopus laevis*. This study further defines the physical and biological properties of this cytokine and demonstrates that TCGF is biochemically similar to mammalian interleukin-2 (IL-2). Biologically active TCGF eluted from SDS-PAGE displays a $M_r$ of 16 kD and lectin-affinity chromatography indicates that the three-dimensionmal configuration of carbohydrates on TCGF and human IL-2 is similar. Secretion of TCGF is detectable 1 day after stimulation of splenocytes with the T-cell mitogen phytohemagglutinin (PHA) and peaks following 2 to 3 days of stimulation. Finally, despite the biological and physical similarities between *Xenopus* TCGF and mammalian IL-2, anti-human IL-2 monoclonal antibodies do not recognize *Xenopus* TCGF.

KEYWORDS: IL-2, *Xenopus*, amphibian, immunity, lymphocyte, cytokine.

### INTRODUCTION

Interleukin-2 (IL-2), one of numerous cytokines released during an immune response, is produced upon stimulation of T cells with antigen (or mitogen) and interleukin-1 (IL-1). Interaction of antigen with the T-cell receptor also induces the expression of IL-2 receptors, thus ensuring antigen-specific T-cell activation (Oppenheim and Gery, 1982). Although IL-2 is primarily associated with T-cell proliferation, it can also directly or indirectly play a role in the proliferation and maturation of other cells. For example, it can induce growth of and immunoglobulin secretion by B cells, evoke superoxide production by activated macrophages, and increase the cytotoxic activity of natural killer cells (Harada et al., 1987; Wahl et al., 1987; Kehrl et al., 1988).

Human IL-2 is secreted as a single polypeptide of 133 amino acids with a molecular mass ($M_r$) of 15.5 kilodaltons (kD) and a single intrachain disulfide bond. The apparent molecular mass heterogeneity reported in early studies results from variable glycosylation and sialylation of the molecule (Robb et al., 1984). IL-2 genes have been cloned from a number of mammalian species including humans (Taniguchi et al., 1983), gibbon (Chen et al., 1985), mice (Yokota et al., 1985), sheep (Seow et al., 1990), pigs (Goodall et al., 1991), and cows (Cerritti et al., 1986), and the molecules they encode exhibit an overall amino acid sequence identity of 50 to 95%. Lymphocyte-derived culture supernatants containing IL-2-like activity have also been described in nonmammalian vertebrates such as fish, frogs, and birds (Cohen and Haynes, 1991).

The anuran amphibian *Xenopus laevis* (South African clawed frog) has an immune system that, in many ways, is comparable to that of mammals. It displays polymorphic class I and class II major histocompatibility complex (MHC) molecules that are involved in allograft rejection, mixed lymphocyte reactions, T–B-cell cooperation and restricted T-cell proliferation, and cytotoxic killing (Du Pasquier et al., 1989; Harding et al., 1993). Additionally, *Xenopus* splenocytes produce an IL-2-like TCGF (Watkins and Cohen, 1987a). *Xenopus* TCGF is produced by T cells and, like mammalian IL-2, promotes growth of activated, but not resting, splenocytes and thymocytes. TCGF is also necessary for the long-term culture of *Xenopus* T-cell lines. There is no apparent *in vitro* crossreactivity between human or mouse IL-

*Corresponding author.

2 and *Xenopus* TCGF (Watkins and Cohen, 1987a) despite their similar biological properties.

This study further defines the physical and biological properties of *Xenopus* TCGF and shows that it displays characteristics comparable to mammalian IL-2. Both the molecular mass of TCGF and its pattern of glycosylation are similar to mammalian IL-2. TCGF production is also similar to mammalian IL-2 in that it is only produced by activated cells (Watkins and Cohen, 1987a) and can be detected in supernatants 1 day after initial stimulation. Finally, even though *Xenopus* TCGF and mammalian IL-2 seem to be homologous, a panel of anti-human IL-2 monoclonal antibodies do not react with *Xenopus* TCGF.

### RESULTS

### Biologically Active TCGF Can Be Eluted from SDS-PAGE

To determine the $M_r$ of the biologically active TCGF protein(s), a saturated ammonium sulfate precipitated TCGF-containing supernatant (SAS-TCGF SN) was run on a 12% nonreducing SDS-PAGE. The gel was then cut into slices, eluted into medium, and assayed for biological activity on splenic blasts. The results of a representative experiment are shown in Fig. 1. Significant biological activity is found in the eluent from slice 9 ($M_r$ >14 and <21 kD), which is similar to human IL-2 (14.6 to 16.2 kD for naturally glycosylated IL-2). By using this method, 40% of the activity found in the starting supernatant (SN) was recovered.

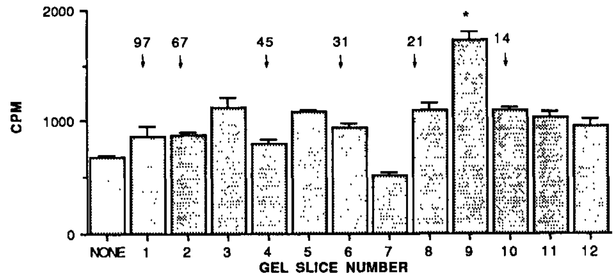

FIGURE 1. Biologically active TCGF can be eluted from SDS-PAGE. SAS-TCGF SN (0.5 ml) was run on a nonreducing 12% SDS-PAGE. The gel was cut into 8-mm slices and protein was eluted into L15 with 0.25% BSA. Each sample was dialyzed with APBS and then assayed on 3-day-old *Xenopus* splenic blasts in a 3-day $^3$H-thymidine-incorporation assay. Because a 10-kD $M_r$ cutoff membrane was used, fractions 11 and 12 serve as additional controls. The data from a representative are expressed as CPM±SE. Numbers above the graph indicate $M_r$ standards in kD. *$p$<0.005 in paired Student's $t$-test.

To determine the $M_r$ of the biologically active molecule(s), eluent from a gel slice containing proteins between 14 and 21 kD was iodinated with $^{125}$I, run on a 12% reducing SDS-PAGE, and autoradiographed. As shown in Fig. 2, this sample contains 16-kD protein. Because protein(s) from this fraction was shown to have biologically activity (Figs 1 and 3) in four separate experiments, we are tentatively naming this 16-kD protein the TCGF molecule.

### Biological Activity of Lectin-Adherent SAS-TCGF Fractions

Glycosylation of human IL-2 with neuraminic acid residues gives rise to peptides with $M_r$ from 14.6 to 16.2 kD (Robb and Smith, 1981). To determine if the *Xenopus* TCGF molecule is similarly glycosylated, affinity chromatography employing wheat germ agglutinin (WGA) and limu-

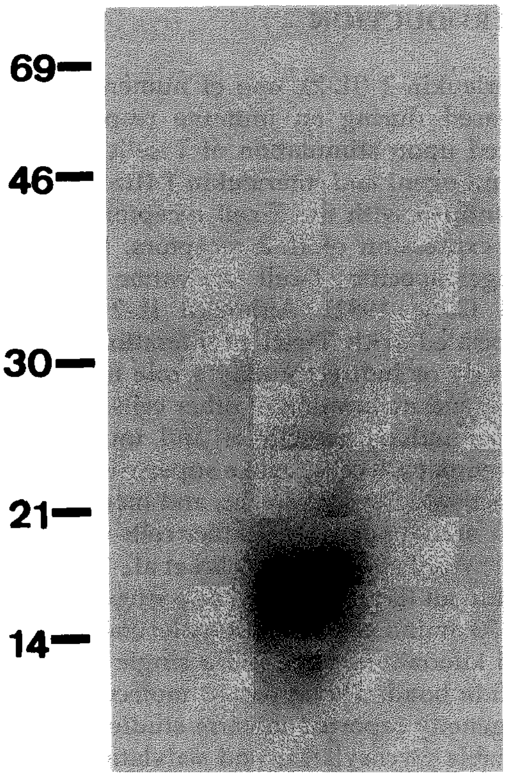

FIGURE 2. Iodination of biologically active gel eluent. Representative autoradiograph of $^{125}$I-labeled biologically active gel eluent run on a 12% reducing SDS-PAGE.

lus lectin (LPA) conjugated to agarose beads was performed with the SAS-TCGF SN. WGA is specific for N-acetyl glucosamine and binds human IL-2; LPA is specific for neuraminic acid and does not bind human IL-2 (Clark-Lewis and Schrader, 1982).

SAS-TCGF SN was applied to each column and incubated on a rocker platform for 60 min at room temperature. Unbound protein was washed off the column and bound protein was specifically eluted with the appropriate buffer. Adherent fractions were concentrated and assayed on splenic blasts. The WGA adherent fraction exhibited biological activity on splenic blasts that was comparable to SAS-TCGF SN (Fig. 4A). The LPA adherent fraction exhibited minimal biological activity (Fig. 4B). Both nonadherent fractions exhibited biological activity (data not shown). It is not known whether this resulted from overloading of the column or the presence of growth factor(s) that cannot bind the lectin. These results with *Xenopus* TCGF are similar to those found with human IL-2 (Clark-Lewis and Schrader, 1982), most likely indicating a similar pattern of glycosylation.

### Lectin Affinity Chromatography of SAS TCGF SN

To determine which protein(s) in the SAS TCGF SN were binding to WGA and LPA lectins, affinity chromatography was performed (as in Fig. 4) with $^{35}$S-methionine-labeled PHA SN. Both the nonadherent (non) and adherent (ad) fractions from the LPA and WGA columns were run on a 12% reducing SDS-PAGE and autoradiographed (Fig. 5).

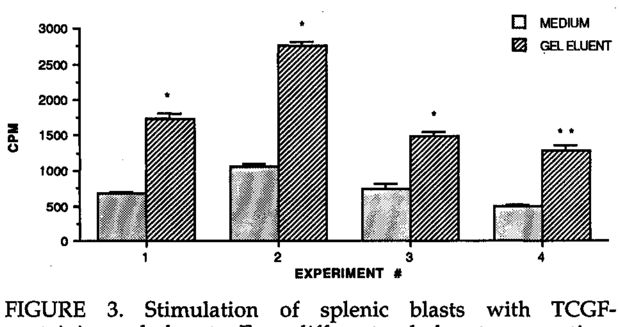

FIGURE 3. Stimulation of splenic blasts with TCGF-containing gel eluents. Four different gel eluent preparations containing the 16-kD TCGF protein were assayed on four separate populations of 3-day-old *Xenopus* splenic blasts in a 3-day $^3$H-thymidine incorporation assay. The data are expressed as CPM±SE. *$p<0.005$, **$p<0.05$ in paired Student's *t*-test.

Both nonadherent fractions are quite similar. They show darker 14-kD (*Xenopus* hemoglobin, data not shown) and fainter 16- and 45-kD bands, as well as IgY heavy chains (69-kD doublet) and light chains (26 and 29 kD) as detected by immunoprecipitation with the mouse anti-*Xenopus* IgY monoclonal antibody XY11.2.2 (Shoemaker et al., 1984) (data not shown). The LPA ad fraction contains the 14-kD band and very faint IgY heavy and light chains. The WGA ad fraction contains the 16-kD putative TCGF protein along with 29- and 42-kD proteins.

### Tunicamycin Treatment

Human IL-2 has an O-linked glycosylation on threonine in amino acid position 3 (Robb et al., 1984). In order to determine if *Xenopus* TCGF is similarly glycosylated, the antibiotic tunicamycin (TM), which blocks the addition of N-linked but has no effect on O-linked glycosylations (Mahoney and Duskin, 1979), was used. $^{35}$S-met labeled PHA SNs were generated from freshly harvested lymphocytes incubated with PHA with or without 5 $\mu$g/ml TM, run on a 12% reducing SDS-PAGE, and autoradiographed. Figure 6

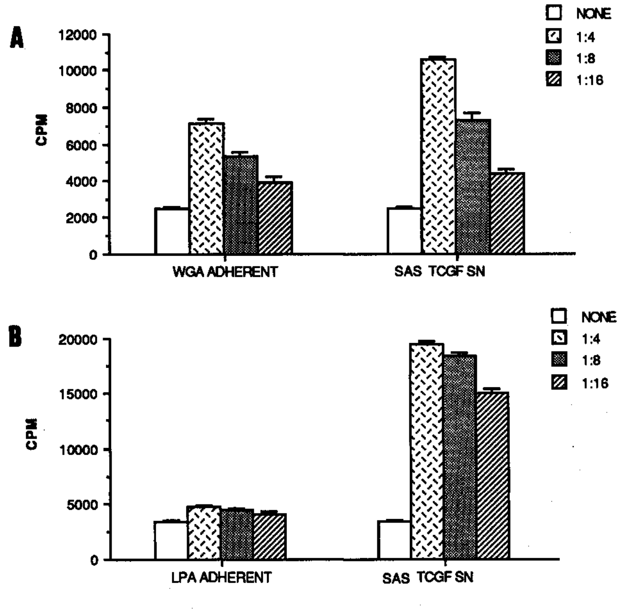

FIGURE 4. Biological activity of lectin adherent SAS-TCGF fractions. WGA (A) and LPA (B) adherent SAS-TCGF fractions (see Materials and Methods) and whole SAS-TCGF SN were assayed for TCGF activity on 3-day-old *Xenopus* splenic blasts in a 3-day $^3$H-thymidine incorporation assay. The data from a representative experiment are expressed as CPM±SE.

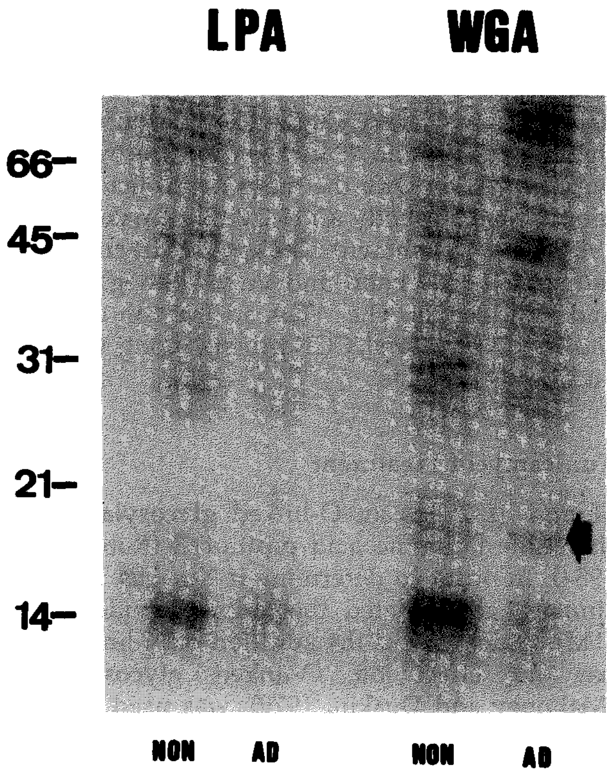

FIGURE 5. Lectin affinity chromatography of radiolabeled PHA SN. Representative autoradiograph of 12% reducing SDS-PAGE of WGA and LPA nonadherent (non) and adherent (ad) fractions (see Materials and Methods) of $^{35}S$ labeled PHA SN. The arrow indicates putative 16-kD TCGF molecule.

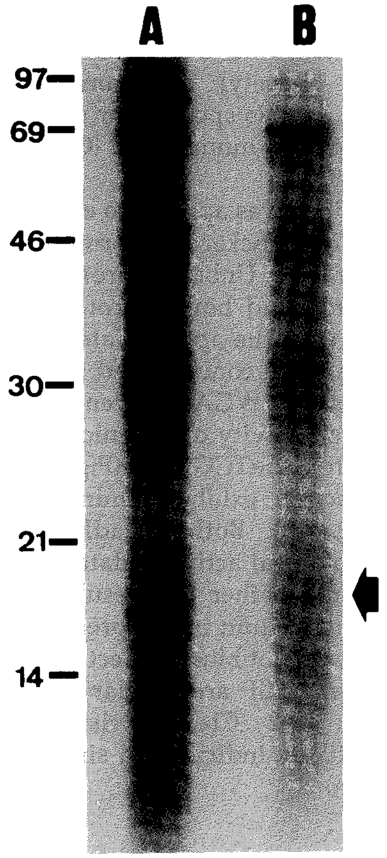

FIGURE 6. Tunicamycin treatment. Representative autoradiograph of 12% reducing SDS-PAGE of $^{35}S$-met labeled PHA SN (lane A) and PHA/TM SN (lane B). The arrow indicates putative 16-kD TCGF molecule.

shows that TM has no effect on the 16-kD TCGF protein in the PHA/TM SN (lane B) because it has the same $M_r$ as the untreated PHA SN (lane A). This indicates that there are no N-linked glycosylations in this protein. Because there are no N-linkages, carbohydrates must be connected via O-linkages as is seen with human IL-2. The PHA SN contains more protein than the PHA/TM SN because TM slows protein synthesis.

### Immunoprecipitation with Anti-Human IL-2 MoAbs

*Xenopus* TCGF is very similar in its physical and biological characterstics to human IL-2, anti-human IL-2 MoAbs (anti-HuIL-2 MoAbs) were used to try to immunoprecipitate TCGF activity from PHA-induced supernatants. Figure 7A shows that the immunoprecipitated *Xenopus* supernatants exhibit stimulatory activity on *Xenopus* thymic blasts that is comparable to untreated supernatant; Fig. 7B reveals that the immunoprecipitated samples of HurIL-2 exhibit a decreased activity on CTLL cells. This experiment indicates that these three anti-HuIL-2 MoAbs do not bind to *Xenopus* TCGF and, therefore, would not be useful in the study or purification of TCGF.

### DISCUSSION

Cytokines are low-molecular-weight peptides that are produced during the course of an immune response and are involved in regulating the intensity and duration of the response (Oppenheim et al., 1991). Some nonmammalian vertebrates can exhibit immune system characteristics similar to mammals (Du Pasquier, 1989), so it is not surprising that these animals also produce cytokines that are analogous to those of

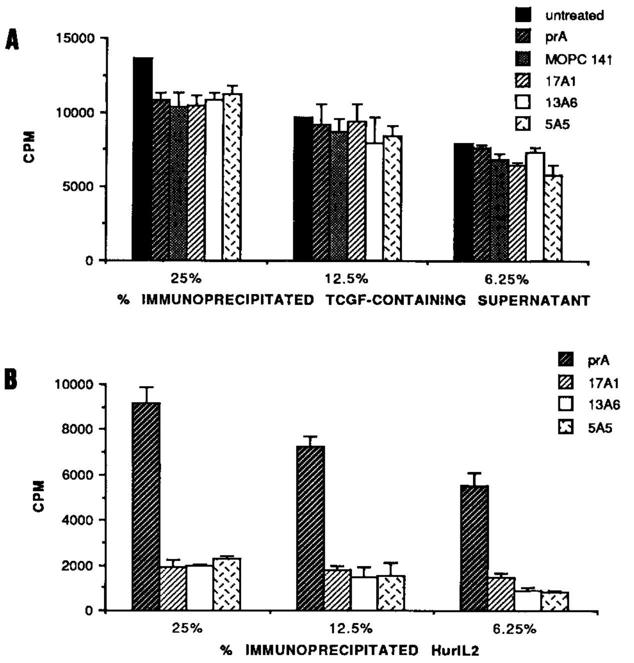

FIGURE 7. Immunoprecipitation with anti-human IL-2 MoAbs. *Xenopus* SAS-TCGF SN and HurIL2 were immunoprecipitated with the anti-HuIL2 MoAbs 17A1, 13A6, and 5A5, or prA beads alone and then assayed on (A) *Xenopus* thymic blasts of (B) murine CTLLs, respectively, in a 3-day $^3$H-thymidine incorporation assay. The results from a representative experiment are expressed as mean CPM±SE. Mean CPM with medium alone are 289±102 for *Xenopus* thymic blasts and 770±172 for CTLLs.

mammals. In fact, T-cell stimulating factors have been described in mitogen- or alloantigen-induced supernatants from fish, frog, and chicken leukocytes (Cohen and Haynes, 1991).

In this report, we have further characterized an IL-2-like molecule from the anuran amphibian, *Xenopus laevis*. Earlier experiments showed that supernatants from splenocytes stimulated with T-cell mitogens, alloantigens, or phorbol esters promoted growth of T-cell blasts and alloreactive *Xenopus* T-cell lines (Watkins, 1985; Watkins et al., 1988), and that TCGF was produced by T cells. TCGF was shown to promote growth of activated, but not resting, adult splenic and thymic T cells (using a monoclonal anti-T-cell antibody developed by Nagata [1985]) and activity could be absorbed by splenic or thymic lymphoblasts but not freshly harvested thymocytes or splenocytes (Watkins and Cohen, 1987b). Additionally, *Xenopus* PHA-induced supernatants are capable of stimulating activated *Xenopus* B cells to proliferate; therefore, it can also function as a B-cell growth factor (Cohen and Haynes, 1991).

The experiments described in this paper demonstrate, by SDS-PAGE analysis, that *Xenopus* TCGF is a secreted peptide of 16 kD. Experiments with lectin affinity chromatography also showed that TCGF binds WGA, much like human IL-2, which has variable O-linked sialylation on the threonine in position three (Robb et al., 1984). It is likely that the carbohydrates on *Xenopus* TCGF are O-linked because tunicamycin, which blocks the addition of N-linked glycosylations (Mahoney and Duskin, 1979), has no effect on TCGF $M_r$. Additionally, TCGF can be detected in radiolabeled SNs 1 day after initial stimulation; secretion peaks at day 3 and begins to decline by day 4 (data not shown).

Although *Xenopus* TCGF and mammalian IL-2 appear biochemically and functionally homologous, *Xenopus* supernatant has no activity on human lymphoblasts or murine HT2 cells and human and murine supernatants and HurIL-2 have no activity on *Xenopus* blasts (Watkins and Cohen, 1987a). Neither has crossreactivity been detected between fish and avian supernatants and mammalian IL-2 (Cohen and Haynes, 1991). Although TCGF preparations also contain B-cell growth-factor activity (Cohen and Haynes, 1991), we have been unable to detect functional crossreactivity between *Xenopus* TCGF and murine IL-4 using the IL-4-dependent cell line CT.4S (Hu-Li et al., 1989) kindly provided by Dr. W. E. Paul at the NIH (data not shown). These results are not surprising because fish, anuran amphibian and chicken immunoglobulins, and MHC molecules also exhibit structural but not functional homology to their mammalian counterparts (Hashimoto et al., 1990; Flajnik et al., 1991, Litman et al., 1991; Miller, 1991). Experiments described in this report also show that several mouse anti-HuIL-2 MoAbs do not bind to *Xenopus* TCGF and, therefore, would not be useful in purification of the TCGF molecule.

The information compiled in this study will be useful in cloning the gene that encodes *Xenopus* TCGF. Because TCGF and IL-2 appear to be biochemically similar, the polymerase chain reaction (PCR) might be employed using oligonucleotide primers derived from the conserved regions of known mammalian IL-2 genes. Alternatively, primers can be derived from the TCGF amino acid sequence, once it is obtained. Attempts to sequence the biologically active gel eluent yielded very little sequence data, indicating that the N-terminal may be blocked. As was the case with mammalian interleukins, the exact nature of TCGF will not be known until the gene coding for the biologically active protein has been cloned.

### MATERIALS AND METHODS

### Animals

Adult female *Xenopus laevis* were purchased from Xenopus I (Ann Arbor, MI) or the African Xenopus Facility (Noordhoek, South Africa). Juvenile *Xenopus* (<1 year old) were bred and maintained in our laboratory according to standardized procedures (Nieuwkoop and Faber, 1967).

### Preparation of Splenic and Thymic Blasts

Splenocytes ($5\times10^6$/ml) were cultured in complete medium [Leibovitz's L-15 medium (Gibco, Grand Island, NY) adjusted to amphibian osmolarity (220 mOsm) and supplemented with $1.25\times10^{-5}$ M HEPES buffer (Gibco), 100 U/ml penicillin, 100 $\mu$g/ml streptomycin (Gibco, Grand Island, NY), $1\times10^{-2}$ M $NaHCO_3$, $5\times10^{-5}$ M 2-mercaptoethanol (Sigma, St. Louis)], with 10% heat-inactivated fetal bovine serum (FBS) (Hyclone, Logan, UT), and 1 to 2 $\mu$g/ml PHA-P (Sigma) for the indicated number of days at 26°C in 24-well plates (Costar, Cambridge, MA). The resulting blasts were then centrifuged (350xg) over Histopaque $\delta$=1.077 (Sigma) and washed twice in complete medium with 1% FBS. Thymocytes were harvested from juvenile *Xenopus* and thymic blasts were generated in a similar manner except 10% ammonium sulfate precipitated PHA-induced supernatant (see what follows) was included in the culture.

### Production of TCGF-Containing Supernatants

Supernatants (SN) were generated as previously described (Watkins and Cohen, 1987a). Briefly, splenocytes from 50 to 70 animals were incubated at $5\times10^6$ cells/ml in complete medium with 0.25% bovine serum albumin (BSA) and 1 $\mu$g/ml PHA-P (Sigma) or 1 to 2 $\mu$g/ml PHA conjugated to agarose beads (Sigma) and the 24 hr and 48 hr supernatants were collected. Soluble PHA was removed from the supernatants by absorption with chicken erythrocytes; PHA beads were removed by centrifugation. The resulting SN was precipitated with saturated ammonium sulfate (SAS-TCGF SN), dissolved in 10% of the original volume, dialyzed (Spectra/Por membrane, $M_r$ cut off 6,000–8,000) with amphibian PBS (APBS) and sterile filtered before use.

### Assay for TCGF Activity

TCGF activity was assyaed on splenic or thymic blasts in a 3-day $^3$H-thymidine incorporated assay. One hundred microliters of the samples to be tested were incubated with $5\times10^4$ blasts (in 100 $\mu$l complete L-15 with 1% FBS) in 96-well round-bottom plates (Costar). After 48 hr, 1 $\mu$Ci/well $^3$H-thymidine (Amersham, Arlington Heights, IL) was added. The cultures were harvested after 72 hr and processed for liquid scintillation spectrometry. All cultures were plated in triplicate and the data are presented as the mean counts per minute (CPM)±SE.

### Production of Radiolabeled Supernatants

Freshly harvested splenocytes from a single animal were centrifuged over Histopaque $\delta$=1.119 (Sigma), washed and resuspended to 5 to $10\times10^6$/ml in complete L15 with 0.25% BSA and 2 $\mu$g/ml PHA. Cells were havested after 2 days, washed twice in sterile APBS (SAPBS) and resuspended in the same volume of complete methionine-free medium (RPMI Select-amine Kit) (Gibco) supplemented with 100 U/ml penicillin, 100 $\mu$g/ml streptomycin $1\times10^{-2}$ M $NaHCO_3$, $5\times10^{-5}$ M 2-mercaptoethanol, 5 $\mu$g/ml insulin, and 5 $\mu$g/ml transferin) with 0.5 mCi $^{35}$S-methionine (Amersham). Supernatants were collected after 24 hr of incubation and concentrated using Centricon Microconcentrators (Amicon, Beverly, MA) with a $M_r$ cutoff of 10,000.

### SDS-Polyacrylamide Gel Electrophoresis (SDS-PAGE)

Twelve percent reducing gels were prepared, run as described by Laemmli (1970) in a vertical gel slab unit (Hoefer Scientific, San Francisco) and stained with 0.25% Coomassie blue. To prepare autoradiographs, gels were soaked for 1 hr in

Autofluor (National Diagnostics, Manville, NJ) and dried. The dried gels were exposed to X-ray film (Kodak X-Omat AR film) at –70°C until the desired intensity was achieved.

### Elution of Biologically Active TCGF from SDS-PAGE

SAS-TCGF SN (0.5 ml) was run on a 10 or 12% nonreducing SDS-PAGE. Prestained rainbow protein molecular weight markers (Amersham) were used to eliminate the Coomassie staining step. The gel was cut into 8-mm slices and eluted into complete L15 with 0.25% BSA for 48 hr. Each sample was then dialyzed in APBS, concentrated using Centricon Microconcentrators with a $M_r$ cutoff of 10,000, and resuspended in a final volume of 0.5 ml L15. Each fraction was assayed for TCGF activity as described before.

### Iodination

Protein from biologically active gel eluent was iodinated with $^{125}I$ (Amersham) using the Enzymobead iodination reagent (Bio-Rad, Richmond, CA) following manufacturer's instructions. After iodination, unincorporated $^{125}I$ was removed by passage over an Exocellulose GF-5 desalting column (Pierce Chemical Co., Rockford, IL), and fractions containing protein were pooled.

### Lectin Affinity Chromatography

Five-milliliter columns were prepared with wheat germ agglutinin (WGA, from *Triticum vulgaris*) or lectin from *Limulus polyphemus* (LPA) conjugated to agarose beads (EY Laboratories, San Mateo, CA). For WGA affinity chromatography, the running buffer was APBS and the elution buffer was 0.1 M N-acetylglucosamine in APBS; for limulus lectin, the running buffer was 0.02 M Tris-HCl, 0.01 M $CaCl_2$, 0.15 M NaCl, pH 7.0, and the elution buffer was 0.02 M Na citrate, 0.15 M NaCl, pH 5.0. Approximately 2 ml of SAS-TCGF SN was applied to each column and incubated on a rocker platform for 40 to 60 min at room temperature. Unbound protein was washed off the column and bound protein was specifically eluted with the appropriate buffer. Protein concentrations were monitored using the Bio Rad protein assay reagent according to manufacturer's instructions. Adherent fractions were concentrated using Centricon Microconcentrators with a $M_r$ cutoff of 10,000, sterile filtered, and assayed for biological activity.

LPA and WGA affinity chromatography was also performed using 24 hr $^{35}S$-labeled PHA supernatants. Two hundred-microliter packed beads, 500-$\mu l$ binding buffer, and 100-$\mu l$ $^{35}S$-labeled PHA supernatant were incubated on a rotator overnight at room temperature. The nonadherent supernatant was collected and the beads were washed four times with the appropriate binding buffer. To collect the adherent fraction, 200 $\mu l$ of elution buffer were added and the samples were rotated for 10 min at room temperature. Both the adherent and nonadherent fractions were then run on 12% reducing SDS-PAGE.

### Immunoprecipitation

One nanomole human recombinant IL-2 (HurIL-2) (Cetus/Perkin Elmer, Norwalk, CT) was immunoprecipitated with 100 $\mu g$ of an anti-HuIL-2 MoAB (17A1, 13A6 or 5A5, kindly provided by Dr. Richard Chizzonite, Hoffman-LaRoche, Nutley, NJ) overnight at 4°C, rotating; 100 $\mu l$ of 50% protein A-Sepharose (prA) beads (Pharmacia, Piscataway, NJ) in 1% FBS-murine PBS was added and incubated for an additional 6 hr at 4°C, rotating. The resulting supernatants were sterile filtered and assayed on mouse CTLL ($5 \times 10^3$ cells/well) in a 3-day $^3H$-thymidine incorporation assay. *Xenopus* SAS-TCGF SN was similarly immunoprecipitated except the subclass control myeloma MOPC 141 (Sigma, St. Louis) was also used. The resulting supernatants were assayed on 8-day-old *Xenopus* thymic lymphocytes ($2 \times 10^4$ cells/well) in a 3-day $^3H$-thymidine incorporation assay.

### ACKNOWLEDGMENTS

We greatly appreciate Dr. Richard Chizzonite of Hoffman-LaRoche for providing the anti-human IL-2 monoclonal antibodies and Dr. W. E. Paul of the NIH for providing the CT.4S cells. We would also like to thank Carolyn Suzuki for performing the immunoprecipitation experiments, Anne Koniski for technical assistance, and David Albright for animal care. Supported by

NIH grants HD-07901 (N.C.) and T32-AI-07285 (F.A.H.).

*(Received January 19, 1993)*

*(Accepted April 7, 1993)*

### REFERENCES

Cerretti D.P., McKereghan K., Larsen A., Cantrell M.A., Anderson D., Gillis S., Cosman D., and Baker P.E. (1986). Cloning, sequence, and expression of bovine interleukin 2. Proc. Natl. Acad. Sci. USA **83:** 3223–3227.

Chen S.J., Holbrook N.J., Mitchell K.F., Vallone C.A., Greengard J.S., Crabtree G.R., and Lin Y. (1985). A viral long terminal repeat in the interleukin 2 gene of a cell line that constitutively produced interleukin 2. Proc. Natl. Acad. Sci. USA **82:** 7284–7288.

Clark-Lewis I., and Schrader J.W. (1982). Biochemical characterization of regulatory factors derived from T cell hybridomas and spleen cells II. Evidence for glycosylation of T cell growth factor, T cell replacing factor, and granulocyte-macrophage colony stimulating factor. J. Immunol. **128:** 175–180.

Cohen N., and Haynes L. (1991). The phylogenetic conservation of cytokines. *In:* Phylogenesis of immune function, Warr G.W., and Cohen N. Eds. (Boca Raton, FL: CRC Press), pp. 241–268.

Du Pasquier L. (1989). Evolution of the immune system. *In:* Fundamental immunology, 2nd ed., Paul W.E. Ed. (New York: Raven Press), pp. 139–165.

Du Pasquier L., Schwager J., and Flajnik M.F. (1989). The immune system of *Xenopus*. Ann. Rev. Immunol. **7:** 251–275.

Flajnik M.F., Canel C., Kramer J., and Kasahara M. (1991). Evolution of the major histocompatibility complex: Molecular cloning of MHC class I from the amphibian *Xenopus*. Proc. Natl. Acad. Sci. USA **88:** 537–541.

Goodall J.C., Enery D.C., Bailey M., English L.S., and Hall L. (1991). cDNA cloning of porcine interleukin 2 by polymerase chain reaction. Biochim. Biophys. Acta **1089:** 257–258.

Harada N., Matsumoto M., Koyama N., Shimizu A., Honju T., Tominaga A., and Takatsu K. (1987). T cell replacing factor/interleukin 5 induces not only B-cell growth and differentiation, but also increased expression of interleukin 2 receptor on activated B-cells. Immunol. Lett. **15:** 205–15.

Harding F.A., Flajnik M.F., and Cohen N. (1993). MHC restriction of T cell proliferative responses in *Xenopus*. Devel. Compar. Immunol. **17:** (in press)

Hashimoto K., Nakanishi T., and Kurosawa Y. (1990). Isolation of carp genes encoding major histocompatibility complex antigens. Proc. Natl. Acad. Sci. USA **87:** 6863–6867.

Hu-Li J., Ohara J., Watson C., Tsang W., and Paul W.E. (1989). Derivation of a T cell line that is highly responsive to IL-4 and IL-2 (CT.4R) and of an IL-2 hyporesponsive mutant of that line (CT.4S). J. Immunol. **142:** 800–807.

Kehrl J.H., Dukovich M., Whalen G., Katz P., Fauci A.S., and Greene W.C. (1988). Novel interleukin 2 (IL-2) receptor appears to mediate IL-2-induced activation of natural killer cells. J. Clin. Invest. **81:** 200–205.

Laemmli W.K. (1970). Cleavage of structural proteins during the assembly of the head of bacteriophage T4. Nature **227:** 680–685.

Litman G.W., Varner J., and Harding F. (1991). Evolutionary origins of immunoglobulin genes. *In:* Phylogenesis of immune function, Warr G.W., and Cohen N. Eds. (Boca Raton, FL: CRC Press), pp. 171–190.

Mahoney W.C., and Duskin D. (1979) Biological activities of the two major components of tunicamycin. J. Biol. Chem. **254:** 6572–6576.

Miller M.M. (1991). The major histocompatibility complex of chickens. *In:* Phylogenesis of immune function, Warr G.W., and Cohen N. Eds. (Boca Raton, FL: CRC Press), pp. 151–169.

Nagata S. (1985). A cell surface marker of thymus-dependent lymphocytes in *Xenopus laevis* is identifiable by mouse monoclonal antibody. Eur. J. Immunol. **15:** 837–841.

Nieukoop P. and Faber J. (1967). Normal table of *Xenopus laevis* (Daudin) (Amsterdam: North Holland).

Oppenheim J.J., and Gery I. (1982). Interleukin 1 is more than an interleukin. Immunol. Today **3:** 113–119.

Oppenheim J.J., Ruscetti F.W., and Faltynik C. (1991). Cytokines. *In:* Basic and clinical immunology, 7th ed., Stites D.P., and Teri A.I. Eds. (Norwalk, CT: Appleton and Lange), pp. 78–100.

Robb R.J., Kutney R.M., Panico M., Morris H.R., and Chowdhry V. (1984). Amino acid sequence and post-translational modification of human interleukin 2. Proc. Natl. Acad. Sci. USA **81:** 6486–6490.

Robb R.J., and Smith K.A. (1981). Heterogeneity of human T-cell growth factor(s) due to variable glycosylation. Mol. Immunol. **18:** 1087–1094.

Robey F.A., and Liu T.Y. (1981). Limulin: A C-reactive protein from *Limulus polyphemus*. J. Biol. Chem. **256:** 969–975.

Seow H.-F., Rothel J.S., Radford A.J., and Wood P.R. (1990). The molecular cloning of the ovine interleukin 2 gene by the polymerase chain reaction. Nucleic Acids Research **18:** 7175.

Shoemaker T.M., Bleicher P.A., and Cohen N. (1984). Studies of the immunoglobulins of the clawed frog *Xenopus*. Dev. Comp. Immunol. Suppl. 3, 156.

Taniguchi T., Matsui H., Fujita T., Takaoka C., Kashima N., Yoshimoto R., and Hamuro J. (1983). Structure and expression of a cloned cDNA for human interleukin 2. Nature **302:** 305–310.

Wahl S.M., McCarthey-Francis N., Hunt D.A., Smith P.D., Wahl L.M., and Katona I.M. (1987). Monocyte interleukin 2 receptor gene expression and interleukin 2 augmentation of microbicidal activity. J. Immunol. **139:** 1342–1347.

Watkins D. (1985). T cell function in Xenopus. Ph.D. dissertation, University of Rochester, Rochester, NY.

Watkins D., and Cohen N. (1987a). Mitogen-activated *Xenopus laevis* lymphocytes produce a T-cell growth factor. Immunology **62:** 119–125.

Watkins D., and Cohen N. (1987b). Description and partial characterization of a T cell growth factor from the frog, *Xenopus laevis*. *In:* Immune regulation by characterized peptides, Goldstein, G., Bach, J.F., and Wigzell H. Eds. (New York: Alan L. Liss), pp. 495–508.

Watkins D., Harding F.A., and Cohen N. (1988). In vitro proliferative and cytotoxic responses against *Xenopus* minor histocompatibility antigens. Transplantation **45:** 499–501.

Yokota T., Arai N., Lee F., Rennick D., Mosmann T., and Arai K.-I. (1985). Use of a cDNA expression vector for isolation of mouse interleukin 2 cDNA clones: Expression of T cell growth factor activity after transfection of monkey cells. Proc. Natl. Acad. Sci. USA **82:** 68–72.

